# Exploring Small-Molecule Inhibitors of Glucosidase II: Advances, Challenges, and Therapeutic Potential in Cancer and Viral Infection

**DOI:** 10.3390/ijms262411867

**Published:** 2025-12-09

**Authors:** Tay Zar Myo Oo, Yupanun Wuttiin, Kanyamas Choocheep, Warunee Kumsaiyai, Piyawan Bunpo, Ratchada Cressey

**Affiliations:** 1 Division of Clinical Chemistry, Department of Medical Technology, Faculty of Associated Medical Sciences, Chiang Mai University, Chiang Mai 50200, Thailand; tayzar_m@cmu.ac.th (T.Z.M.O.); kanyamas.c@cmu.ac.th (K.C.); warunee.kumsaiyai@cmu.ac.th (W.K.); piyawan.b@cmu.ac.th (P.B.); 2 Division of Transfusion Sciences, Department of Medical Technology, Faculty of Associated Medical Sciences, Chiang Mai University, Chiang Mai 50200, Thailand; yupanun.wuttiin@cmu.ac.th

**Keywords:** glucosidase II, N-linked glycoprotein, cancer, *PRKCSH*, ER, glycosylation

## Abstract

Glucosidase II (GluII) is a heterodimeric enzyme localized in the endoplasmic reticulum (ER), essential for the sequential trimming of glucose residues during N-linked glycosylation. This critical function facilitates glycoprotein folding via the calnexin/calreticulin chaperone system, maintaining ER homeostasis. Dysregulation or inhibition of GluII has been implicated in various pathological processes, including cancer, viral infections, and glycoprotein misfolding disorders. This review summarizes the current knowledge of GluII’s structure and function, highlights a wide range of natural and synthetic GluII inhibitors—including iminosugar derivatives (e.g., deoxynojirimycin (DNJ), castanospermine (CAST)), non-iminosugar compounds (e.g., bromoconduritol, catechins), and mechanism-based cyclophellitol analogues—and evaluates their biological effects and therapeutic potential. The cellular impact of GluII inhibition is explored in the context of ER stress, unfolded protein response (UPR), tumor cell apoptosis, and viral replication. Key challenges in developing selective GluII inhibitors are discussed, with a focus on strategies to minimize off-target effects, including prodrug design, allosteric modulation, and emerging genetic approaches such as microRNA (miRNA)-mediated downregulation of GluII subunits. Taken together, these insights underscore the therapeutic relevance of GluII as a druggable target and pave the way for the rational design of next-generation inhibitors in oncology, infectious diseases, and metabolic disorders.

## 1. Introduction

ER Glucosidase II (GluII) is a heterodimeric enzyme containing of a catalytic α subunit (104–116 kDa) and a regulatory β subunit (58–80 kDa), encoded from *PRKCSH* (protein kinase C substrate 80 K-H) [1,2,3,4]. The α subunit, part of the glycosidase hydrolase family 31, performs the enzyme’s catalytic function, while the β subunit ensures ER localization via a HDEL (His-Asp-Glu-Leu) retention motif [1,2,5]. Structurally, the α subunit includes four major domains, among which the B1 subdomain may facilitate α/β interaction [6]. Although GluII predominantly resides in the ER, evidence suggests that it may also localize to other cellular compartments [7].

Functionally, GluII catalyzes the sequential removal of glucose residues from the N-linked oligosaccharide Glc_3_Man_9_GlcNAc_2_ to yield Man_9_GlcNAc_2_ [8,9,10]. This trimming process is essential for proper protein folding and quality control within the ER, as it governs glycoprotein interaction with the chaperones calnexin and calreticulin, ensuring correct folding and ER homeostasis [11]. Misfolded glycoproteins are re-glucosylated by UDP-glucose glycoprotein glucosyl transferase and repeatedly processed until proper folding is achieved [12,13]. Hence, GluII exerts a dual regulatory function—facilitating initial folding through chaperone exposure while ensuring exit from the folding cycle through final glucose removal [11].

Dysregulation of GluII disrupts this delicate balance, contributing to multiple diseases. Overexpression of GluII has been reported in urothelial carcinoma [14], gastric cancer [15] and melanoma [16], where it promotes cancer cell survival and poor prognosis [17]. Conversely, inhibition or deletion of GluII results in the accumulation of misfolded glycoproteins, activation of the unfolded protein response (UPR), and induction of ER stress [4]. Genetic mutations in PRKCSH cause polycystic liver disease by impairing glycoprotein processing and altering cellular proliferation and differentiation [18]. Moreover, many enveloped viruses—such as flaviviruses, coronaviruses, and influenza viruses—rely on GluII for correct folding of viral glycoproteins; inhibition of GluII disrupts viral maturation and significantly reduces infectivity [19,20,21,22]. Knockout of the GluII β-subunit has also been shown to suppress lung cancer cell growth by reducing receptor tyrosine kinase signaling [23].

Given its central role in glycoprotein maturation, ER homeostasis, and disease pathology, GluII represents a promising therapeutic target. Pharmacological inhibition of GluII offers potential for the development of novel anticancer and antiviral therapies, as well as interventions for glycoprotein-related disorders. This review provides a comprehensive overview of ER GluII, with emphasis on its structure, biological functions, and therapeutic relevance. Section 2 discusses the enzyme’s molecular architecture and catalytic mechanism. Section 3 explores different classes of GluII inhibitors, including natural and synthetic compounds, and their structure–activity relationships. Section 4 examines the biological and clinical implications of GluII inhibition, focusing on cancer and viral infections, while Section 5 highlights challenges, knowledge gaps, and perspectives for future drug development.

By integrating structural biology, biochemical mechanisms, and pharmacological evidence, this review aims to elucidate how GluII functions as a key regulator of ER proteostasis and how its inhibition can be strategically harnessed in disease treatment. The implications extend beyond enzyme inhibition itself—shedding light on the broader concept of targeting ER quality-control machinery as a novel therapeutic avenue.

The search strategy and criteria used in this review are summarized in Table 1.

## 2. Glucosidase II: Structure and Function

### 2.1. Molecular Structure

**Figure 1:**
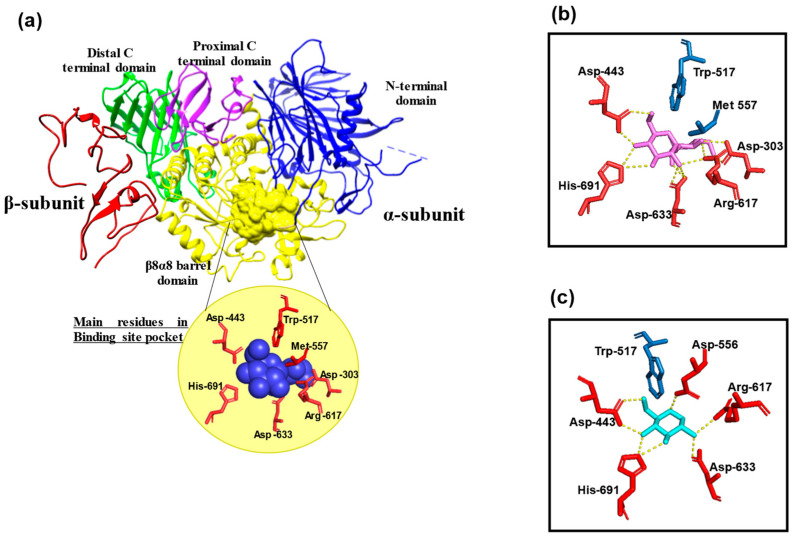
Visualization of binding site pocket in GluII enzyme and comparison of key residue interactions with substrate and inhibitor. (**a**) Ribbon structures of GluII enzyme (PDB 5JQP): The N-terminal domain and β8α8 barrel domain of α-subunit are depicted in blue and yellow, respectively. The proximal and distal C terminal domains of α-subunit are shown in purple and green colors. The β-subunit is represented in red color. The binding site pocket is illustrated as yellow surface, while the substrate or inhibitor is represented as a blue sphere. (**b**) Substrate (Glc1Man2))-GluII interaction and (**c**) Inhibitor (DNJ)-GluII interaction in binding site pocket: Yellow dashed lines show hydrogen bond interactions. Ligands and residues involved in binding sites are illustrated in stick models.

GluII is a soluble heterodimeric enzyme composed of a catalytic α-subunit (GluII-α) and a regulatory β-subunit (GluII-β), as identified through genetic and biochemical studies. The α-subunit, homologous to other glucosidases, carries the catalytic function, while the β-subunit is responsible for ER retention via its HDEL signal and does not contribute catalytically [1,2,3,4]. These two subunits interact non-covalently to form a stable heterodimeric complex [1]. Studies using *Schizosaccharomyces pombe* have demonstrated that both subunits are essential for enzymatic activity, because GluII becomes inactive in the absence of genes encoding either subunit [4]. The overall ribbon structure of the GluII heterodimer, highlighting the α- and β-subunits, is illustrated in Figure 1.

The structure of GluII-α comprises four major domains (N-terminal, β8α8 barrel, proximal C-terminal, and distal C-terminal) and three subdomains (B1, B2, and B3). The N-terminal domain forms a β-sandwich with additional α-helices and a conserved N-loop that contributes to the active site pocket [6,24,25,26,27,28,29,30]. The β8α8 barrel domain, making up ~40% of the protein, contains the core catalytic site, stabilized by the proximal and distal C-terminal domains. Subdomain B1, located in the N-terminal domain, interacts with the β subunit and exhibits flexibility due to disordered residues (215–235). Subdomains B2 and B3, in the β8α8 barrel domain, have unique structural features, with B3 lacking defined secondary structures.

The β subunit contains an HDEL motif, which facilitates retention in the ER via interaction with the KDEL (Lys-Asp-Glu-Leu) receptor [2,31,32,33]. It also contains two EF-hand domains that bind calcium ions, along with negatively charged amino acid repeats that enhance calcium-binding activity [34,35,36]. Two functional domains, ID1 (Immunoglobulin-like domain 1) and ID2, enable interactions with the α subunit. ID1, located at the N-terminal region, contains cysteine-rich elements but forms no disulfide bonds, supporting non-covalent linkages [1,37]. ID2, located in the 273–400 residue region, is proline-rich and contains a critical binding region of 127 residues, with alternative splicing in seven amino acids not affecting binding [38]. The simultaneous binding of α and β subunits increases the stability of the complex, ensuring functionality and retention within the ER [37].

Glucosidase I and II belong to different glycoside hydrolase (GH) families (GH63 and GH31, respectively) and have distinct roles in the N-glycan processing pathway as well as clearly different molecular architectures. While the active site of glucosidase I is relatively accommodating and flexible, which reflects its larger, more complex structure, glucosidase II has a more constrained active site shaped by specific polypeptide segments known as exclusion loops. These loops narrow the active-site pocket, so inhibitors must be carefully optimized in size and conformation to achieve high GluII affinity. GluII also contains an insertion between the +1 and +2 subsites that contributes to its catalytic activity and substrate specificity. Structural studies indicate that inhibitors targeting the conserved ring of aromatic residues located between the +1 and +2 subsites may provide increased selectivity and potency for GluII. In contrast, glucosidase I specificity is guided by the unique conformation of its large, branched substrate Glc_3_Man_9_GlcNAc_2_; computational analyses suggest that the active site pocket is tailored to accommodate the Glc_3_ residue, creating a distinctive binding pocket that can be exploited by selective inhibitors. Table 2 summarizes the key molecular differences between glucosidase I and II, including the structural features underlying their distinct inhibitor specificities [39,40,41,42].

### 2.2. Function

**Figure 2:**
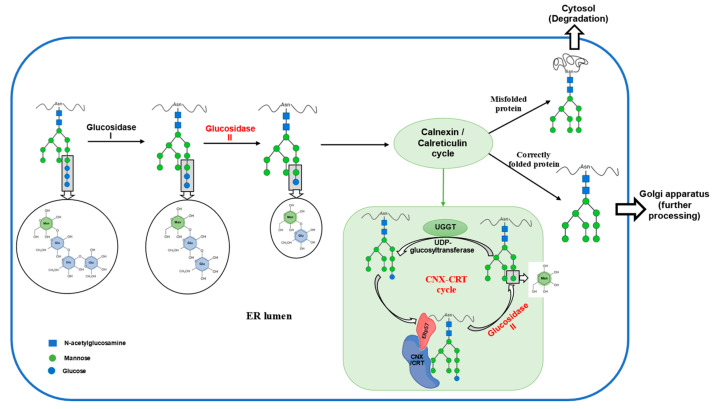
Schematic of the calnexin/calreticulin (CNX/CRT) cycle: sequential trimming by glucosidase I and glucosidase II generate G1M9, chaperone binding supports folding, UGGT-mediated re-glucosylation returns misfolded clients to the cycle, and correctly folded proteins exit to the Golgi for further processing; persistently misfolded proteins are targeted for degradation. (Symbols: blue squares, N-acetylglucosamine; green circles, mannose; blue circles, glucose).

GluII is central to N-glycan trimming during protein folding. It sequentially removes glucose residues from Glc2Man9GlcNAc2 (G2M9) to produce monoglucosylated glycans (Glc1Man9GlcNAc2 or G1M9), which interact with ER chaperones calnexin and calreticulin to prevent aggregation and facilitate proper folding [8,11]. Re-glucosylation by UDP-glucose glycoprotein glucosyltransferase (UGGT) allows for iterative folding attempts until proteins achieve their correct conformation [12]. Once folding is complete, GluII removes the final glucose, enabling the glycoprotein to exit the folding cycle and proceed along the secretory pathway [11]. The overall quality-control loop is depicted in Figure 2.

Structural studies have shown that GIIα’s catalytic mechanism involves key residues, including Asp556 and Asp633, which drive glycan hydrolysis [6]. The β subunit’s MRH (Mannose-6-phosphate receptor homology) domain further enhances substrate binding, supporting efficient glycan processing. In vivo studies using mutant cells lacking GIIβ reveal delayed glycan trimming and disrupted protein maturation [4,43]. The β subunit’s HDEL motif ensures GluII remains localized within the ER, maintaining proper enzymatic function [1,4]. Thus, GluII’s coordinated activity is central to glycoprotein processing, ER homeostasis, and cellular protein quality control.

## 3. Glucosidase II Inhibitors

Glucosidase II inhibitors that target the glucosidase enzyme involved in trimming glucose residues from N-linked glycoproteins have gained attention for their therapeutic potential, particularly in treating viral infections, cancer, diabetes, and lysosomal storage disorders [20,44,45,46]. This section focuses on the structure of glucosidase II inhibitors, their mechanisms of inhibition, and their structural interactions with the enzyme’s active site.

**Figure 3:**
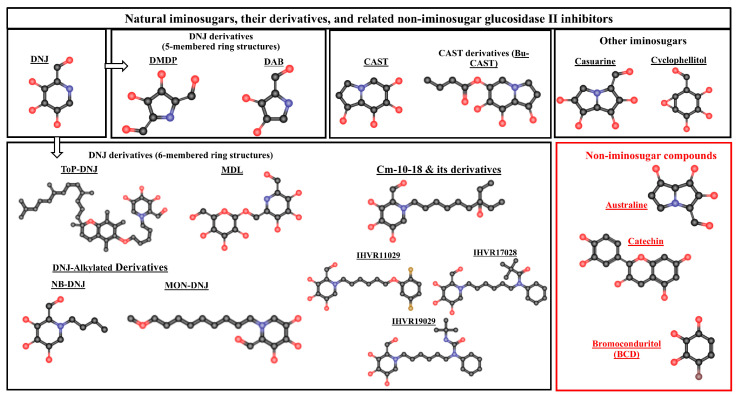
Structures of natural iminosugars, their derivatives, and related non-iminosugar glucosidase II inhibitors. Color code for atomic representation: Dark grey dots: carbon (C) atoms forming the backbone of the ring structures and side chains; red dots: oxygen (O) atoms present in hydroxyl (–OH) and hydroxymethyl (–CH_2_OH) groups; blue dots: nitrogen (N) atoms incorporated into the ring structures; Hydrogen atoms are omitted for clarity.

Glucosidase II inhibitors can be classified into three major categories based on their origin and chemical nature: natural iminosugars and their derivatives, non-iminosugar natural products, and synthetic cyclophellitol-based inhibitors. Each class exhibits unique structural features that influence their selectivity and inhibitory mechanisms (Figure 3).

Natural Glucosidase II inhibitors are classified into two major groups: iminosugars and non-iminosugar compounds. Left and upper panels: show natural iminosugars and their derivatives, including DNJ (1-deoxynojirimycin) and its analogs. DNJ derivatives are subdivided into five-membered ring structures such as DMDP (deoxymannojirimycin) and DAB (deoxygalactonojirimycin), and six-membered ring structures such as ToP-DNJ (5′-tocopheroxypentyl-DNJ) and MDL (2,6-dideoxy-2,6-imino-7-O-(β-D-glucopyranosyl)-D-glycero-L-guloheptitol). Further modifications of DNJ, including alkylated derivatives (NB-DNJ (N-butyl-1-deoxynojirimycin) and MON-DNJ (N- (9′-methoxynonyl)-deoxynojirimycin)), improve lipophilicity and cell permeability. CM-10-18 and its analogs (IHVR1029, IHVR7028, and IHVR9029) are synthetic DNJ-based derivatives designed to enhance inhibitory potency and selectivity toward Glucosidase II. Top-right panels: depict CAST (castanospermine) and its butylated derivative (Bu-CAST (6-O-Butanoyl castanospermine)), as well as other iminosugars such as casuarine and cyclophellitol, which share a polyhydroxylated piperidine or cyclitol core conferring competitive inhibition of ER α-glucosidases. Right panel, red box: shows non-iminosugar inhibitors, including australine (an azasugar alkaloid), catechin (a polyphenolic compound that modulates glycosidase activity indirectly through redox signaling), and BCD (bromoconduritol), a halogenated cyclitol derivative reported to inhibit Glucosidase II activity through a non-classical mechanism.

### 3.1. Natural Iminosugars and Their Derivatives

Iminosugars are polyhydroxylated alkaloids with a nitrogen atom substituting for the oxygen atom typically found in sugars. This structural change allows iminosugars to mimic the glycosidic transition state and competitively bind to the active site of glucosidase II, inhibiting its function [47,48]. Their nitrogen atom stabilizes the positive charge of the oxycarbenium ion intermediate, enhancing inhibition.

#### 3.1.1. Deoxynojirimycin (DNJ)

Nojirimycin is the first glucose analog characterized by a nitrogen atom replacing the oxygen atom in its structure, enhancing its biological activity by mimicking glucose and effectively inhibiting various glucosidases. This natural compound is found in strains of *Streptomyces, Bacillus*, and mulberry tree leaves and has been recognized as a potent glucosidase inhibitor. However, its structural instability, primarily due to the presence of a hydroxyl (-OH) group at the C-1 position, limits its practical application. The reduced form, deoxynojirimycin (DNJ), addresses this limitation and serves as a more stable and effective glucosidase inhibitor. DNJ acts as a competitive inhibitor by binding to the active site of the glucosidase enzyme. It effectively inhibits glucosidase activity in both yeast (*S. cerevisiae*) and IEC-6 intestinal epithelial cells [49]. DNJ demonstrates greater efficiency in inhibiting glucosidase II (GluII) compared to glucosidase I (GluI), as 2 µM of DNJ is sufficient to inhibit GluII, whereas 20 µM is required to inhibit GluI. This significant difference suggests that DNJ preferentially blocks GluII activity, leading to the accumulation of partially trimmed oligosaccharides. These observations are derived from in vitro studies using purified glucosidases from *Saccharomyces cerevisiae* and microsomal preparations, which demonstrate the direct biochemical effects of DNJ on glucosidase enzymes. In calf pancreas microsomes, DNJ inhibited glucosidase activity in enzyme assays, and in IEC-6 intestinal epithelial cell cultures DNJ was shown to impair the formation of complex N-linked oligosaccharides [49]. When DNJ inhibits GluII, the oligosaccharide trimming process is halted prematurely, resulting in the accumulation of partially processed oligosaccharides with fewer than three glucose residues. This disruption can impair the proper folding and quality control of glycoproteins within the ER.

In untreated control cells, the predominant oligosaccharide precursor for glycoprotein synthesis is Glc_3_Man_9_(GlcNAc)_2_-PP-dolichol. However, in IEC-6 cells treated with DNJ, the precursor shifts to Man_9_(GlcNAc)_2_-PP-dolichol. This suggests that DNJ interferes with the initial attachment of the oligosaccharide to asparagine residues on nascent proteins, potentially affecting N-linked glycosylation and the overall maturation of glycoproteins [50]. By examining the crystal structures of α-glucosidase II in complex with DNJ, it is evident that the endocyclic nitrogen atom of DNJ closely interacts with D564 and occupies the -1 subsite. This interaction mimics the natural glucose substrate, effectively inhibiting the enzyme reaction by preventing further catalytic activity [41].

DNJ is a valuable tool for scientists investigating the role of glucose residues in glycoprotein synthesis, folding, and quality control. By selectively inhibiting glucosidase II, DNJ alters the composition of oligosaccharides and disrupts the glycosylation process, affecting proper protein maturation. As a result, DNJ provides critical insights into glycoprotein biochemistry and holds promise as a potential therapeutic candidate for diseases involving glycosylation defects.

#### 3.1.2. DNJ Derivatives (6-Membered Ring Structure)

1. ToP-DNJ4 (DNJ-tocopherol conjugate)

Deoxynojirimycin (DNJ) was chemically linked to α-tocopherol, a non-toxic form of vitamin E that is naturally absorbed through the gut and directed to the liver, where it accumulates within the membranes of immune cells. The resulting iminoglycoside derivative, known as 5′-tocopheroxypentyl-DNJ (ToP-DNJ), demonstrates selective binding to endoplasmic reticulum glucosidase II (ER GluII) over other glucosidases, including those found in the intestinal lumen [51,52,53,54]. In vitro observations revealed that even at a relatively high concentration (50 µM), ToP-DNJ inhibited less than 50% of intestinal glucosidase activity, thereby minimizing the risk of gastrointestinal side effects. ToP-DNJ exhibited an IC_50_ value of 9 µM against ER GluII using a pNPG substrate, comparable to DNJ (13 µM) and NB-DNJ (miglustat, 16 µM) [55]. Its high specificity for ER GluII is attributed to the aromatic tocopherol moiety, which interacts with a hydrophobic exclusion loop near the enzyme’s active site—a structural feature unique to GluII.

The in vitro inhibition of GluII by ToP-DNJ was confirmed in cellular assays, with its effectiveness varying by cell type. GluII inhibition was observed only in myeloid lineage cell lines (e.g., monocyte-derived macrophages (MDMΦ) and HL60), while Huh7.5, Jurkat, and Raji cells showed no effect, as demonstrated by accumulation of mono-/di-glucosylated glycans in free-oligosaccharide (FOS) assays. Consistent with this, ToP-DNJ exhibited antiviral activity in infected MDMΦ cells, inhibiting dengue virus replication with an IC_50_ of 12.7 µM, as measured by tissue culture infectious dose 50% (TCID50) assays [55]. In vivo studies showed that despite its poor oral bioavailability, ToP-DNJ selectively accumulates in the liver, achieving better liver and plasma distribution compared to miglustat, a currently approved iminosugar [55]. This liver-targeting property is attributed to its α-tocopherol moiety, which naturally directs it to the liver upon absorption.

Given its enzyme, cell, and tissue selectivity, ToP-DNJ holds promise as an antiviral agent, particularly for diseases like dengue and hepatitis, where liver and immune cells play key roles. Conjugation of iminosugars with native metabolites, such as tocopherol, may further enhance their selectivity and therapeutic potential for various diseases [55].

2. Alkylated derivatives of DNJ (NB-DNJ and MON-DNJ)

NB-DNJ (N-butyl-1-deoxynojirimycin), known as miglustat, and MON-DNJ (N-(9′-methoxynonyl)-DNJ), also called UV-4, are synthesized derivatives of 1-deoxynojirimycin (DNJ). NB-DNJ is formed by alkylating DNJ with a butyl group, while MON-DNJ is synthesized via alkylation with a nonyl halide. The key structural difference lies in their alkyl chains: NB-DNJ has a butyl group, while MON-DNJ features a longer, more hydrophobic nonyl group [41]. A unique structural feature in ER α-GluII, the F607 residue in the exclusion loop, confers specificity to these derivatives, reducing off-target effects. The butyl tail of NB-DNJ displaces the side chain of W525 in the +1 subsite, causing disorder and allowing interactions with residues like F307 and F571. The longer nonyl tail of MON-DNJ extends further into the +1 and +2 subsites, enhancing binding through hydrophobic interactions with F307 and F571. This longer chain also induces significant conformational changes in the α523-528 loop, making it unstable and enhancing inhibition [41].

MON-DNJ exhibits stronger inhibition than DNJ and NB-DNJ, as seen through its lower IC_50_ values against isolated Mmα-GluII [41]. Therapeutically, miglustat (NB-DNJ) is used to treat Gaucher disease [20] and non-insulin-dependent diabetes [56], while MON-DNJ has demonstrated in vivo antiviral activity against dengue virus [57], influenza A [58] and influenza B [48] with its antiviral efficacy against influenza B being the first reported case [48].

3. 2,6-dideoxy-2,6-imino-7-O-(beta-D-glucopyranosyl)-D-glycero-L-guloheptitol (MDL)

MDL is an iminosugar derivative containing a nitrogen atom in its ring and a β-D-glucopyranosyl group attached to its backbone. Structurally designed to mimic natural substrates of glucosidase enzymes, MDL effectively binds and inhibits their activity. Initially developed to target intestinal glucohydrolase and sucrase [59], MDL also inhibits other enzymes, including maltase, isomaltase, glucoamylase, trehalase, and to a lesser extent, lactase and β-amylase [60].

Although no in silico studies have been performed on MDL with glucosidase enzymes (including GluI and GluII), in vitro studies indicate its preference for inhibiting glucosidase II. MDL selectively interferes with the later steps of glucose trimming in the ER while having minimal effect on glucosidase I activity. At 250 µg/mL, MDL blocked the removal of glucose residues from N-linked glycoproteins of influenza virus, leading to the accumulation of incomplete oligosaccharides (Glc_2_Man_7_-_9_GlcNAc), indicating potent inhibition of glucosidase II. At higher concentrations (500 µg/mL), both glucosidase I and II were affected, demonstrating dose-dependent selectivity [60].

Importantly, MDL does not significantly impact protein or RNA synthesis or lipid-linked oligosaccharide production, supporting its specificity for glycoprotein processing. Its selective inhibition of GluII and interference with viral glycoprotein maturation highlight MDL’s potential as a tool compound and antiviral agent.

4. CM10-18 and its derivatives

CM10-18, a derivative of OSL-95II (a modified 1-deoxynojirimycin iminosugar), features an opened cyclohexyl ring on its N-linked side chain, enhancing its antiviral activity and toxicity [61]. In vivo, CM10-18 treatment results in elevated Glc_1_Man_4_GlcNAc_1_ levels, indicating inhibition of glucosidase activity during glycan trimming. However, the absence of free oligosaccharides suggests limited inhibition of α-glucosidase I, with retained dose-dependent selectivity for glucosidase II [62].

CM10-18 functions similarly to NBDNJ, selectively inhibiting GluII and disrupting glycoprotein maturation—a mechanism underpinning its antiviral activity. It shows potent inhibition of dengue virus (DENV) by blocking glycoprotein folding and secretion of infectious particles [61,62]. To expand its antiviral spectrum, a structure–activity relationship (SAR) study of 120 derivatives identified IHVR11029, IHVR17028, and IHVR19029, which exhibited broad-spectrum in vitro activity against all four viral families causing viral hemorrhagic fevers (VHFs). These compounds significantly reduced mortality in Marburg (MARV) and Ebola (EBOV) virus-infected mice [47].

These findings underscore the therapeutic potential of iminosugar-based GluII inhibitors for treating viral infections.

#### 3.1.3. DNJ Derivatives (5-Membered Ring Structure)

1. 2,5-dideoxy-2,5-imino-D-mannitol (DMDP)

DMDP is a pyrrolidine-based iminosugar containing two hydroxymethyl and two hydroxyl groups. It occurs naturally in *Derris elliptica* leaves and *Lonchocarpus sericeus* seeds and can also be synthesized chemically. As a structural analog of β-D-fructofuranose, DMDP mimics natural glucosidase substrates and acts as a competitive inhibitor [63,64]. DMDP binds glucosidases, including GluII, through ionic interactions, although it lacks stabilizing hydrogen bonds, making it less effective than CAST or Casuarine (CSU) in silico [65]. Nonetheless, it inhibits glucose trimming in the ER and disrupts both GluI and GluII, with stronger inhibition of GluI, resulting in the accumulation of Glc_3_Man_9_GlcNAc_2_—indicative of early-stage glycoprotein processing disruption [63].

Structural modifications affect DMDP’s potency: substitution with fluorine, amino, or methoxy groups, or changes at the hydroxymethylene carbon, reduce activity [65,66], while lipophilic modifications enhance efficacy [67]. Its inhibitory activity is also pH-dependent, with reduced function in acidic conditions, similar to swainsonine and castanospermine. DMDP is reported to be 10–60 times more potent than DNJ, and has potential as an anti-HIV agent through inhibition of glycoprotein folding [68]. Due to its toxicity and antifeedant effects, it may also be applied as a natural insecticide [64].

2. 1,4-dideoxy-1,4-imino-D-arabinitol (DAB)

DAB is a natural pyrrolidine-based iminosugar isolated from *Angylocalyx botiquenus* [39]. It acts as a competitive inhibitor of GluII, forming ionic bonds with Asp546 and Asp640, which enhances binding affinity, as shown in in silico modeling [69]. In enzyme assays, DAB was found to be 10-fold more potent than comparable compounds, effectively inhibiting glucose trimming in ER-derived microsomes.

DAB also exhibits inhibitory activity against several mammalian enzymes, including α-mannosidases I and II, intestinal isomaltase, and trehalas [68]. Although it has been evaluated as an anti-HIV agent, its antiviral efficacy is limited, suggesting that GluII inhibition alone may not suffice for strong antiviral activity [69]. Nonetheless, enhancing its GluII selectivity offers potential for antiviral drug development, similar to ToP-DNJ [55].

#### 3.1.4. Castanospermine (CAST)

CAST is a bicyclic iminosugar (tetrahydroxyoctahydro-indolizidine) isolated from *Castanospermum australe* (the Australian chestnut tree). Its gluco-type stereochemistry mimics glucose, allowing it to act as a competitive inhibitor of both α- and β-glucosidases, including GluI and GluII, which are essential for N-linked glycoprotein processing [70,71,72,73]. Crystal structure studies show that CAST’s endocyclic nitrogen forms a key interaction with Asp564 in the catalytic site of GluII, while its unique hydrophobic ring fits into a conserved pocket formed by W423, I448, and W525, enhancing binding affinity and inhibitory potency [41].

Functionally, CAST disrupts glycoprotein maturation, delaying ER-to-Golgi transport of LDL and insulin receptors [74,75]. In vivo, it slows tumor growth and reverses cancer cell transformation by inhibiting the maturation of oncogenic glycoproteins [76]. In HIV, it impairs viral replication and syncytium formation, supporting its role as an antiviral agent [77,78,79]. Overall, CAST holds promise as both an anticancer and antiviral agent by selectively targeting glucosidase II and interfering with glycoprotein processing.

#### 3.1.5. Castanospermine Derivatives (Celgosivir, Bu-Cast)

Bu-Cast (6-O-Butanoyl Castanospermine) is a lipophilic derivative of CAST, featuring a butanoyl group at the 6th position. Celgosivir, its prodrug, shares the same structure and is metabolized into castanospermine in vivo [80,81]. Both act as competitive inhibitors of α-glucosidase I and II, binding the catalytic site of GluII and disrupting glycoprotein folding [41,73]. Celgosivir inhibits dengue virus (DENV) replication by accumulating misfolded NS1 proteins in the ER, thereby blocking their transport to the Golgi. This impairs viral assembly and triggers unfolded protein response (UPR), enhancing host antiviral defenses. NS1 misfolding is especially detrimental, as this protein is critical for DENV replication and pathogenesis.

In vitro, celgosivir demonstrates broad-spectrum activity against all four DENV serotypes (DENV1–4) at low concentrations, showing greater efficacy than its parent compound [82]. It also exhibits antiviral effects against HIV by disrupting glycoprotein processing, with Bu-Cast showing stronger inhibition of GluI in culture models [81]. Celgosivir advanced to Phase II clinical trials for hepatitis C virus (HCV) and HIV, highlighting its potential as a CAST-derived antiviral agent [83,84].

#### 3.1.6. Other Iminosugars

1. Casuarine (CSU)

CSU is a naturally occurring bicyclic pyrrolizidine iminosugar isolated from *Casuarina equisetifolia* (Australian pine) and *Eugenia jambolana* (Java plum) [85]. Its structure includes a pyrrolizidine backbone, a hydroxymethyl group at position 3, and multiple stereospecific hydroxyl groups. CSU binds the active site of GluII through ionic interaction with Asp564 and hydrogen bonding with His698, enhancing binding stability and inhibitory potency [39]. In silico and enzyme assays show CSU is 4–7 times more effective than DMDP, with moderate potency compared to other iminosugars (DAB > Cast > CSU > DMDP). It inhibits GluII more strongly than GluI and effectively blocks glucose trimming in ER microsomes, regardless of glycan status.

Recent studies show CSU also inhibits glucoamylase and, in derivative form, trehalase Tre37A, indicating potential for enzyme-targeted therapeutics [86]. Given its origin from medicinal plants with known antidiabetic and anticancer properties [85,87]. CSU holds promise for therapeutic development, particularly for diabetes and cancer, through structural optimization for selective enzyme inhibition.

2. Cyclophellitol-type compounds

Cyclophellitol is a natural product derived from *Phellinus* species, known for its mechanism-based inhibition of β-glucosidase [88]. To target α-glucosidases, structural modifications are required to improve selectivity and stability. Notably, α-1,2- and α-1,5a-cyclophellitols, synthetic analogues with six-membered cyclitol rings and epoxide groups at different positions, bind covalently to α-glucosidases via nucleophilic attack by catalytic residues, leading to irreversible enzyme inactivation [89,90,91,92,93,94].

Comparative studies show that α-1,5a-cyclophellitol analogues exhibit stronger inhibitory activity than α-1,2 analogues against GluII and recombinant human acid α-glucosidase (rhGAA). Furthermore, N-alkyl-1,5a-aziridine, a derivative of α-1,5a-cyclophellitol, selectively inhibits GluII in the ER, showing five-fold greater selectivity over rhGAA [95]. To reduce off-target effects, modifications such as replacing the epoxide with a cyclic sulfate have been developed. The 1,6-epi-cyclosulfate derivative demonstrates superior GluII inhibition and effectively blocks viral replication, outperforming other iminosugars and cyclophellitol analogues [40,96]. Overall, cyclophellitol-based inhibitors—especially those structurally optimized—offer a promising, selective approach for studying GluII function and developing therapeutics targeting GluII-related diseases.

### 3.2. Non-Iminosugar Compounds

#### 3.2.1. Australine

Australine is a naturally occurring tetrahydroxy pyrrolizidine alkaloid isolated from the seeds of *Castanospermum australe*. Its bicyclic structure consists of two fused five-membered rings with a bridging nitrogen atom and hydroxyl groups at positions 1, 2, 3, and 7. Unlike many known glucosidase inhibitors (e.g., CAST, DNJ) that have six-membered rings, australine demonstrates that a five-membered ring structure can also confer potent glucosidase inhibitory activity [97].

Australine acts as a competitive inhibitor of α-glucosidase, showing strong inhibition at low concentrations. It does not affect α/β-mannosidases or galactosidases, indicating its substrate-specific binding. Studies in mung bean seedlings show strong inhibition of glucosidase I and only weak activity against GluII, leading to the accumulation of Glc_3_Man_7_–_9_(GlcNAc)_2_—evidence of disrupted early N-linked glycoprotein processing. In virus-infected Madin–Darby Canine Kidney (MDCK) cell cultures, australine inhibits glycoprotein maturation in a dose-dependent manner, supporting its potential as a therapeutic agent for viral infections and glycosylation-related disorders [98].

#### 3.2.2. Polyphenolic Catechins in Green Tea

Green tea catechins are polyphenolic compounds with natural antioxidant properties. Their structure includes two benzene rings (A and B), a dihydropyran C-ring with two chiral centers, and adjacent phenolic hydroxyl groups forming a catechol moiety [98,99]. The major catechins are EGCG (epigallocatechin-3-gallate), Epigallocatechin (EGC), Epicatechin-3-gallate (ECG), and Epicatechin (EC), with EGCG being the most abundant (50–70%). Unlike others, EGCG contains a fourth aromatic ring (D), contributing to enhanced activity [99,100,101,102]. Docking studies show EGCG inhibits α-glucosidase by forming strong hydrogen bonds via its D-ring with Asp1279, while interactions with Asp1157 on the A-ring region stabilize binding. Its T-shaped conformation enhances binding affinity at the enzyme’s active site [103].

In rat liver microsomes, tea catechins inhibited GluII activity in a concentration-dependent manner, interfering with glycoprotein processing. Based on IC_50_ and K_i_ values, they act as potent GluII inhibitors [104]. These findings suggest that green tea catechins, particularly EGCG, may exert anticancer effects through glucosidase II inhibition, supporting their potential use as dietary supplements or adjuncts in cancer prevention and therapy.

#### 3.2.3. Bromoconduritol (BCD)

Bromoconduritol (6-bromo-3,4,5-trihydroxycyclohex-1-ene; BCD) is a non-iminosugar cyclitol derivative identified as a selective inhibitor of GluII. Early kinetic studies using GluII purified from rat liver revealed that BCD selectively interacts with the low-affinity binding site for maltose substrates, disrupting hydrolysis while sparing high-affinity substrate interactions. These findings led to the development of a dual-site kinetic model describing GluII’s substrate recognition and catalytic behavior [105].

Further biochemical analyses confirmed that BCD inhibits both the cleavage-1 and cleavage-2 steps of glucose trimming in the endoplasmic reticulum, with a marked preference for cleavage-2—the removal of the innermost glucose residue from N-linked glycans. This step is essential for proper glycoprotein folding and quality control. The inhibition was shown to be irreversible, as enzymatic activity did not recover after dialysis [106].

Functional studies in lung carcinoma cells demonstrated that pharmacological or genetic inhibition of the GluII β-subunit results in increased autophagy and p53-dependent apoptosis, supporting a regulatory role for GluII in cell survival and stress response pathways. These observations further underscore the therapeutic relevance of GluII inhibition in oncology [17].

Taken together, BCD represents a mechanistically distinct GluII inhibitor with irreversible activity and selective cleavage-site targeting. Its unique mode of action provides a valuable scaffold for probing GluII biology and for the rational design of next-generation glycosidase inhibitors with clinical potential. The inhibitory potency and biological activities of the glucosidase II inhibitors discussed above are summarized in Table 3. Clinical studies evaluating miglustat and celgosivir across different disease contexts are summarized in Table 4.

## 4. Impact and Therapeutic Implications of Glucosidase II Inhibition

### 4.1. Impact on Protein Folding and ER Stress

**Figure 4:**
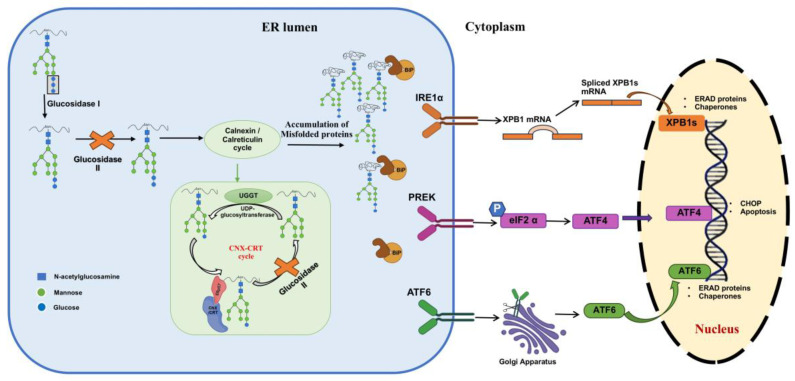
Role of glucosidase II on protein folding and ER stress: Inhibition of GluII prevents the necessary glucose trimming. As a result, monoglucosylated glycans, which are required for binding to the calnexin/calreticulin chaperones, are not formed. This leads to the accumulation of misfolded proteins within the ER lumen, identifiable by their unprocessed glycans (Glc_2_Man_9_GlcNAc_2_). The accumulation of misfolded proteins disturbs ER homeostasis and triggers the Unfolded Protein Response (UPR). The UPR is initiated by the dissociation of the chaperone BiP from three ER stress sensors: IRE1, PERK, and ATF6. Initially, the UPR aims to restore ER homeostasis by suppressing protein synthesis, enhancing chaperone production, and promoting ER-associated degradation (ERAD). However, prolonged or severe ER stress, such as that induced by persistent GluII inhibition, can shift the UPR towards activating apoptotic pathways, ultimately leading to cell death. (Symbols: blue squares, N-acetylglucosamine; green circles, mannose; blue circles, glucose.).

GluII plays a critical role in N-linked glycosylation by trimming glucose residues from nascent glycoproteins in the ER. This glucose removal is essential for the entry of glycoproteins into the calnexin/calreticulin chaperone cycle, which ensures proper protein folding. The role of GluII in protein folding, ER stress, and UPR activation is illustrated in Figure 4. Inhibition of GluII blocks glucose trimming, preventing the formation of monoglucosylated glycans required for chaperone binding. As a result, misfolded proteins accumulate due to improper folding. Studies on GluII-α and GluII-β mutants show complete loss of GluII activity and the absence of monoglucosylated oligosaccharides, accompanied by ER accumulation of misfolded proteins and increased expression of BiP, an ER stress marker [4]. The GANAB gene, encoding the GluII-α subunit, is essential for protein folding and ER stress regulation [120,121]. Accumulated misfolded proteins retain unprocessed glycans (e.g., Glc_2_Man_9_GlcNAc_2_), disturbing ER homeostasis and triggering the unfolded protein response (UPR). UPR is activated via Binding immunoglobulin protein (BiP) dissociation from three ER stress sensors: Inositol-requiring enzyme 1 (IRE1), Protein kinase RNA-like endoplasmic reticulum kinase (PERK), and Activating transcription factor 6 (ATF6) [122,123,124,125]. Initially, UPR suppresses protein synthesis and enhances chaperone production and ER-associated degradation (ERAD). However, prolonged ER stress shifts UPR toward apoptosis [126]. GluII inhibition has been shown to induce autophagy and apoptosis in tumor cells [17,127] highlighting its potential in therapeutic strategies that exploit ER stress-mediated cell death.

### 4.2. Role of Glucosidase II in Cancer

**Figure 5:**
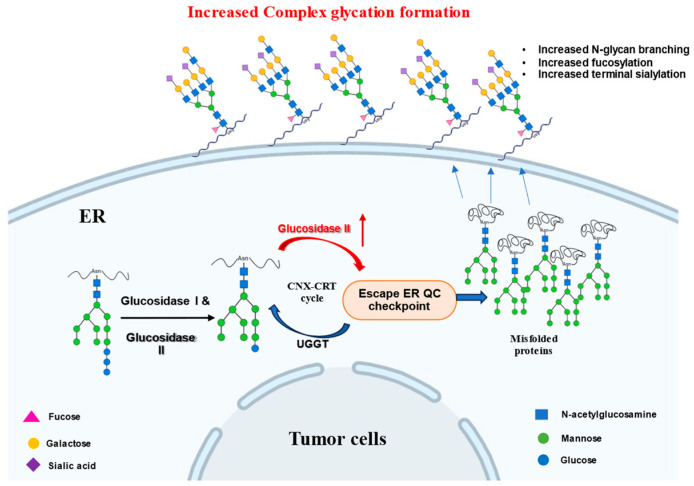
Glucosidase II overactivity on cancer cells: In tumor cells, Glucosidase II (Glu II, also known as PRKCSH) exhibits overactivity, indicated by the red arrow. This heightened activity may contribute to an accelerated and sometime erroneous trimming of N-glycans, hindering the proper folding of glycoproteins. Consequently, a large number of proteins may become misfolded and accumulate in the ER. Despite their misfolded state, it has been suggested that these proteins, often with improperly processed N-glycans, bypass quality control mechanisms (due to the overactive Glu II disrupting the UGGT-mediated re-glucosylation and refolding cycle) and are secreted out of the ER. These misfolded glycoproteins are then aberrantly expressed on the cell surface. The persistent presence of these misfolded proteins on the cell surface of tumor cells has been proposed to influence cancer development, including altered cell signaling, immune evasion, and enhanced proliferation, potentially driving tumor progression. These observations are primarily supported by in vitro studies [17,23,135] and the direct in vivo evidence linking GluII overactivity to tumor progression still remains limited. (Symbols: blue squares, N-acetylglucosamine; green circles, mannose; blue circles, glucose; pink triangles, fucose; yellow circles, galactose; purple squares, sialic acid).

Disruption of ER protein folding—such as through glucosidase II (GluII) inhibition—induces ER stress, activating the unfolded protein response (UPR) to restore homeostasis. However, prolonged or severe ER stress can shift UPR toward mitochondrial apoptosis [128]. The proposed mechanisms by which GluII contributes to cancer cell survival, ER stress adaptation, and tumor progression are illustrated in Figure 5. GluII is essential for protein transport from the ER. In cancer cells, overexpression of GluII may allow escape from ER stress-induced apoptosis, promoting survival under proteotoxic conditions. Therefore, inhibition of GluII may restore ER stress sensitivity, reducing proliferation and inducing apoptosis—a promising anticancer strategy [104,129]. In nude mice bearing EHS-BAM tumors, treatment with the GluI/II inhibitor castanospermine (CAST) significantly suppressed tumor growth, likely by impairing angiogenesis. In C57/BL mice, CAST reduced angiogenic response to basic fibroblast growth factor (bFGF), particularly by inhibiting endothelial cell invasion and migration, without affecting proliferation or extracellular matrix (ECM) attachment. CAST also disrupted the formation of essential cell surface oligosaccharides, impairing tumor vascularization and progression [81,130]. CAST further reduced platelet aggregation linked to metastasis [131], increased myeloma cell adhesion to endothelial cells [132] and impaired integrin-mediated binding in colon carcinoma cells, limiting metastatic potential [133].

In urothelial carcinoma, high GluII expression correlates with tumor grade. GANAB (α-subunit) knockdown reduced proliferation and migration via G1 cell cycle arrest [14]. Additionally, these findings highlight GluII as a therapeutic target in cancer via modulation of glycoprotein processing, ER stress, and tumor progression pathways. Additionally, PRKCSH (β-subunit) knockdown triggered autophagy and apoptosis [17,134]. Subsequent studies demonstrated that GluIIβ loss suppresses tumor growth and metastasis by inhibiting receptor tyrosine kinase signaling [23] and modulates cell adhesion molecule (CAM) expression and anti-tumor immune pathways [135].

Collectively, these findings highlight GluII as a multifunctional regulator in cancer biology, linking glycoprotein processing, ER stress, autophagy, and tumor progression—underscoring its potential as a therapeutic target.

### 4.3. Glucosidase II Inhibition in Infectious Diseases

N-linked glycosylation is essential for viral glycoprotein folding, trafficking, and receptor binding. Since viruses lack glycosylation machinery, they rely on host enzymes such as GluII for proper protein maturation [136,137]. Inhibition of GluII disrupts glucose trimming in the ER, leading to accumulation of Glc_2_Man_9_GlcNAc_2_, which binds to malectin and activates the unfolded protein response (UPR). Misfolded glycoproteins are retained in the ER and directed to ERAD or, in some cases, escape with compromised functionality [138,139,140,141]. In dengue virus (DENV) infection, GluII inhibition misfolds non-structural protein 1 (NS1), envelope protein (E), and pre-membrane protein (prM), triggering UPR and reducing viral replication [82,142]. Similar effects are observed in Zika Virus (ZIKV), Yellow Fever Virus (YFV), and Hepatitis C Virus (HCV), with CRISPR/Cas9 GluII knockout reducing intracellular viral RNA and secretion by 10–100 fold [143]. Modified GluII-selective inhibitors also show potent anti-DENV activity. [55,144,145].

In Severe Acute Respiratory Syndrome Coronavirus 2 (SARS-CoV-2), GluII inhibition disrupts spike protein folding, blocking viral maturation and replication [40]. In influenza virus, GluII inhibitors such as N-nonyl-deoxynojirimycin (NN-DNJ) impair glycosylation of hemagglutinin (HA) and neuraminidase (NA), reducing surface expression and sialidase activity, leading to strain-specific antiviral effects [146,147]. For HIV, GluII inhibition reduces envelope protein (gp120, gp160) expression and disrupts gp120–CD4 binding, impeding infectivity [77,78,148]. Treatment with Bu-CAST in CD4^+^ cells further blocks glycoprotein processing and reduces binding capacity with infected cells [149]. These findings highlight GluII as a promising antiviral target, offering broad-spectrum activity by disrupting viral glycoprotein maturation and host–virus interactions.

## 5. Challenges and Strategies for Selective Glucosidase II Inhibition

Developing selective inhibitors for GluII remains challenging due to the structural similarities among glucosidases. Many inhibitors, such as iminosugars, lack specificity, leading to off-target inhibition of other glycosidases and complicating therapeutic applications [55]. For instance, miglustat, a broad-spectrum glucosidase inhibitor, causes gastrointestinal side effects like diarrhea and weight loss by interfering with intestinal enzymes [150]. To improve selectivity, targeting unique allosteric sites on ER-resident α-glucosidases has been proposed. Unlike conserved active sites, allosteric regions may allow selective modulation of GluII without interfering with intestinal glucosidases. This approach has also been explored in diabetes therapy, where selective inhibition of gut maltase helped minimize adverse effects [151]. Another promising strategy involves prodrug modification. For instance, ester derivatives of DNJ have shown reduced gastrointestinal toxicity by limiting interaction with intestinal disaccharidases [152].

Importantly, genetic approaches, such as miRNA-mediated suppression, offer an emerging alternative. In polycystic liver disease, miR-345 has been shown to reduce proliferation, possibly by downregulating GluII subunits like *GANAB* which encodes the α-subunit, supporting the concept of post-transcriptional regulation as a selective therapeutic strategy [153].

Although there has been significant progress in developing potent GluII inhibitors, further work is still required to improve their selectivity, pharmacological robustness, and safety profile. Future perspectives in GluII-targeted therapy will likely depend on integrating structure-based drug design, ER-targeted delivery systems, and gene-regulatory approaches such as miRNA-mediated modulation of GluII subunits. Together, these strategies hold promise for generating highly selective, effective, and well-tolerated GluII inhibitors applicable to a range of diseases, including cancer, viral infections, and metabolic disorders.

Despite these advances, several critical gaps remain before GluII inhibitors can be translated convincingly into the clinic. First, in vivo quantification of the therapeutic window and pharmacokinetics for the most active analogs—such as epi-cyclophellitol cyclosulfates and advanced DNJ derivatives—has not yet been systematically defined. Second, comprehensive toxicity profiling, including chronic exposure and organ-specific safety studies, is still lacking and will be essential for long-term use. Third, detailed pharmacokinetic/pharmacodynamic characterization is needed to clarify tissue distribution, metabolic stability, and target engagement, and to distinguish on-target from off-target effects, particularly toward non-ER glucosidases. Addressing these issues should be considered a priority for the field, as they represent key steps toward the rational development of GluII-targeted therapeutics for cancer, viral infections, and metabolic diseases.

## Figures and Tables

**Table 1:** Search Strategy and Method.

Parameter	Description
Databases search	PubMed, Web of science, Google scholar
Period cover	1990–2024
Search Terms	Glucosidase, ER Glucosidase II, PRKCSH, Glycoprotein folding, Iminosugars, DNJ, Inhibitors, cancer, virus, Clinical trials, Treatment
Inclusion criteria	Journals focusing on GluII structure, function and inhibitions of the compounds
Exclusion criteria	Non-English papers, unrelated glycosidases, papers lacking primary data

**Table 2:** Key molecular differences between Glucosidase I and II.

Features	Glucosidase I	Glucosidase II
GH Family	GH Family 63	GH Family 31
Structure	Single-pass type II transmembrane protein	Heterodimer composed of a catalytic α-subunit and an accessory β-subunit
Biological substrate	Glc3Man9GlcNAc2	Glc2Man9GlcNAc2, Glc1Man9GlcNAc2
Cleavage	Selectively performs the first trimming step, removing first glucose from Glc3Man9GlcNAc2	Removes glucoses from Glc2Man9GlcNAc2, Glc1Man9GlcNAc2, performing the second and third cleavage steps
Catalytic residues	2 carboxylic residues (general acid & base)	2 carboxylic residues (Nucleophile & General Acid/Base)
Key specificity	Specificity is guided by the unique conformation of the substrate	An insertion between +1 and +2 subsites establishes its activity and substrate specificity
Implication for inhibitors selective design	Targeting the unique pocket specific for the Glc_3_ conformation may help avoid off-target interactions.	Targeting the conserved ring of aromatic residues between the +1 and +2 subsites may yield increased potency and selectivity

**Table 3:** Source, IC_50_ value and biological effects for various inhibitors of glucosidase II.

Inhibitors	Source	In Vitro IC_50_ (µM)	In Vitro Biological Effects	In Vivo/Model Efficacy
1-Deoxynojirimycin (DNJ)	*Streptomyces* and *Bacillus* strains; *mulberry (Morus)* leaves	11.4 µM [21]	Blocks maturation of N-linked glycoproteins—potentially impairing asparagine (N-linked) glycosylation—and preferentially inhibits GluII over GluI.	No specified data in vivo
Castospermine	Seeds of *Castanospermum australe* (Moreton Bay chestnut)	5–8 µM [60]	Inhibits overall glucose trimming (GluI and/or GluII) in ER-derived microsomes and strongly inhibits GII activity.	Improved survival in a dengue mouse model after intraperitoneal dosing at 10, 50, or 250 mg/kg/day for 10 days; the protective effect has not been conclusively attributed to GluII [107].
Bu-CAST (Celgosivir)	Semisynthetic derivative of castanospermine	1.1 µM [108]	Inhibits both GluI and GluII, disrupting protein folding by binding to the catalytic site of GluII.	In a lethal DENV mouse model, it is rapidly metabolized to castanospermine, and its protective efficacy correlates with inhibition of α-glucosidases I and II; the most effective regimen was 50 mg/kg twice daily for 5 days [109].
ToP-DNJ4 (DNJ−tocopherol conjugate)	Derivatives of 1-deoxynojirimycin (DNJ)	9.0 µM [55]	Inhibits GluII activity and mitigates side effects through conjugation with an aromatic tocopherol moiety.	No in vivo data reported
NB-DNJ (N-butyl-1-deoxy nojirimycin)	Alkylated DNJ derivatives	5.2 µM [21]	Inhibits glucosidase activity through dual-site binding; its unique exclusion loop confers higher selectivity for GluII.	Improved survival and reduced viral load in a lethal dengue mouse model; target enzyme not confirmed The most effective dose (Intraperitoneal injection) was 1000 mg/kg per days for 7 days [110].
MON-DNJ (N-(9′-methoxynonyl)-DNJ	Alkylated DNJ derivatives	1.8 µM [22]	Inhibits glucosidase activity with greater potency than other iminosugars such as DNJ and NB-DNJ.	Increased survival (90–100%) in a lethal DENV mouse model at 20 mg/kg TID [22].
2,6-dideoxy-2,6-imino-7-0-(~-D-glucopyranosyl)-D-glycero--L-guloheptitol (MDL)	Derivatives of 1-deoxynojirimycin (DNJ)	1 µM [60]	Disrupts N-glycan processing, impairs glycoprotein maturation, and exhibits potent inhibitory activity against GluII.	No in vivo data reported
CM-10-18	Semisynthetic DNJ derivative (OSL-95II modification)	1.55 µM [62]	Significantly disrupts glucosidase activity in glucose trimming and exhibits dose-dependent selectivity for GluII.	Reduced peak viremia in DENV-infected mice with oral dosing; combination with ribavirin enhanced antiviral activity, with validated targeting of ER α-glucosidase II. Effective regimen: 75 mg/kg every 12 h for 3 days [62]
IHVR-11029	Semisynthetic derivatives of CM-10-18	0.09 µM [47]	Significantly disrupts the protein-folding process through CM-10-18 derivatives, whose enhanced inhibitory effect supports their potential therapeutic application.	Significantly reduced mortality in lethal MARV and EBOV mouse models via inhibition of ER α-glucosidases I and II. Oral gavage: 32 mg/kg for MARV and 25 mg/kg for EBOV every 12 h for 10 days post-infection [47].
IHVR-17028	Semisynthetic derivatives of CM-10-18	0.24 µM [47]	Significantly disrupts the protein-folding process through CM-10-18 derivatives, whose enhanced inhibitory effect supports their potential therapeutic application.	Significantly reduced mortality in lethal MARV and EBOV mouse models via inhibition of ER α-glucosidases I and II. Oral gavage: 32 mg/kg for MARV and 25 mg/kg for EBOV every 12 h for 10 days post-infection [47].
IHVR-19029	Semisynthetic derivatives of CM-10-18	0.48 µM [47]	Significantly disrupts the protein-folding process through CM-10-18 derivatives, whose enhanced inhibitory effect supports their potential therapeutic application.	Significantly reduced mortality in lethal MARV and EBOV mouse models via inhibition of ER α-glucosidases I and II. Oral gavage: 32 mg/kg for MARV and 25 mg/kg for EBOV every 12 h for 10 days post-infection [47].
2,5-dideoxy-2,5-imino-D-mannitol (DMDP)	Leaves of *Derris elliptica* and seeds of *Lonchocarpus sericeus*	No IC_50_ data reported. [111]	More strongly inhibits the early glucose-trimming stage, showing greater effect on GluI than on GluII.	No in vivo data reported
1,4-dideoxy-1,4-imino-D-arabinitol (DAB)	*Angylocalyx spp*. (reported as “A. botiquenus”)	No IC_50_ data reported. [112]	Inhibits glucosidase enzymes, including GluII, and disrupts protein folding.	No in vivo data reported
Casuarine (CSU)	*Casuarina equisetifolia* (Australian pine) and leaves of Java plum Syzygium cumini [*syn. Eugenia jambolana*]	No IC_50_ data reported. [86]	Exhibits stronger inhibition on GluII than on GluI and disrupts the glucose-trimming process.	No in vivo data reported
1,2-cyclophellitol analogues	Semisynthetic cyclophellitol analogue; parent compound isolated from *Phellinus* sp.	11.3 µM [95]	Shows reduced inhibitory activity compared with the 1,5a analogue.	No in vivo data reported
1,5a-cyclophellitol analogues	Semisynthetic cyclophellitol analogue; parent compound isolated from *Phellinus sp.*	0.028 µM [95]	Exhibits better inhibitory properties than the 1,2 analogue and blocks protein folding.	No in vivo data reported
1,6-epi-cyclophellitol cyclosulfate	Semisynthetic cyclophellitol analogue; parent compound isolated from *Phellinus sp.*	0.03 µM [40]	Inhibits GluII activity and reduces viral replication.	No in vivo data reported
Australine	Seeds of *Castanospermum australe*	5.8 µM [98]	Exhibits stronger inhibitory activity on GluI than on GluII and prevents the N-linked glycosylation process.	No in vivo data reported
Polyphenolic catechins -EGCG	Leaves of tea plant (*Camellia sinensis*)	50.92 µM/47.72 µM [104]	Inhibits GluII activity, thereby affecting glycoprotein maturation and quality control in the ER.	Inhibited postprandial blood-glucose rise in mice via intestinal α-glucosidase inhibition, not ER glucosidase. Oral dose: catechin mixture (main monomers) at 50 mg/kg body weight [113].
Polyphenolic catechins -EGC	Leaves of tea plant (*Camellia sinensis*)	117.7/110.5 µM [104]	Inhibits GluII activity, thereby affecting glycoprotein maturation and quality control in the ER.	Inhibited postprandial blood-glucose rise in mice via intestinal α-glucosidase inhibition, not ER glucosidase. Oral dose: catechin mixture (main monomers) at 50 mg/kg body weight [113].
Polyphenolic catechins -ECG	Leaves of tea plant (*Camellia sinensis*)	15.14/19.06 µM [104]	Inhibits GluII activity, thereby affecting glycoprotein maturation and quality control in the ER.	Inhibited postprandial blood-glucose rise in mice via intestinal α-glucosidase inhibition, not ER glucosidase. Oral dose: catechin mixture (main monomers) at 50 mg/kg body weight [113].

**Table 4:** Clinical trial summary of miglustat and celgosivir.

Drug	Disease	Phase	Key Results	Limitations	References
Miglustat	Gaucher disease type 1	Long-term extension study (First Trial)	Reduced spleen volume (30%) and liver volume (18%) over 12 months. Improvement in hemoglobin and platelet count	Diarrhea, weight lost and peripheral neuropathy	[114]
Miglustat	Niemann-Pick Disease Type C (NP-C)	Randomised controlled study (12-month duration)	Beneficial effect on neurological progression over 12 months.	Small number of participants and adverse effect such as diarrhea.	[115]
Miglustat	HIV infection	Phase II (Double-blind, randomized, controlled study)	Suppression of HIV p2 Antigenemia was lower in the combination therapy with zidovudine and increase in CD4 cells was noted.	Gastrointestinal symptoms (diarrhea, abdnominal pain), weigh lost.	[116]
Celgosivir	Dengue virus	Phase 1b (Randomised, double-blind, placebo-controlled, proof-of-concept trial.	No significant efficiency resulted for the primary end points but it was generally safe and well tolerated	Lack of efficiency and side-effects (diarrhea)	[117]
Celgosivir	HCV infection	Phase II trial	Synergistic effect in combination therapy	Stopped in the Migenix financial report for 2010	[118,119]

## Data Availability

No new data were created or analyzed in this study.

## Abbreviations

The following abbreviations are used in this manuscript:

AGIs	α-Glucosidase inhibitors
ATF6	Activating transcription factor 6
BCD	Bromoconduritol
BiP	Binding immunoglobulin protein
Bu-Cast	6-O-Butanoyl castanospermine
CAST	Castanospermine
CNX/CRT	Calnexin/Calreticulin chaperone system
CSU	Casuarine
DAB	1,4-Dideoxy-1,4-imino-D-arabinitol
DEGs	Differentially expressed genes
DENV	Dengue virus
DNJ	Deoxynojirimycin
DMDP	2,5-Dideoxy-2,5-imino-D-mannitol
EBOV	Ebola virus
EC	Epicatechin
ECG	Epicatechin gallate
EGC	Epigallocatechin
EGCG	Epigallocatechin-3-gallate
ER	Endoplasmic reticulum
ERAD	ER-associated degradation
GANAB	Gene encoding the GluII α-subunit
GIIα	Glucosidase II α-subunit
GIIβ	Glucosidase II β-subunit
GluI	Glucosidase I
GluII	Glucosidase II
HCV	Hepatitis C virus
HDEL	His-Asp-Glu-Leu ER-retention motif
HIV	Human immunodeficiency virus
HOXA1	Homeobox A1
IRE1	Inositol-requiring enzyme 1
KDEL	Lys-Asp-Glu-Leu ER-retention motif
MARV	Marburg virus
MDL	2,6-dideoxy-2,6-imino-7-O-(β-D-glucopyranosyl)-D-glycero-L-guloheptitol
miRNA	microRNA
MON-DNJ	N-(9′-methoxynonyl)-deoxynojirimycin
MRH domain	Mannose-6-phosphate receptor homology domain
mTOR	Mechanistic target of rapamycin
NB-DNJ NN-DNJ	N-butyl-1-deoxynojirimycin N-nonyl-deoxynojirimycin
NSCLC	Non-small cell lung cancer
OR	Odds ratio
pNPG	p-Nitrophenyl β-D-glucopyranoside
*PRKCSH* PERK	Protein kinase C substrate 80K-H (Gene encoding the GluII ***β***-subunit) Protein kinase RNA-like endoplasmic reticulum kinase
qPCR	Quantitative polymerase chain reaction
rhGAA	Recombinant human acid α-glucosidase
TCID50	Tissue culture infectious dose 50%
ToP-DNJ	DNJ-tocopherol conjugate
UGGT	UDP-glucose:glycoprotein glucosyltransferase
UPR	Unfolded protein response
VHFs	Viral hemorrhagic fevers
YFV	Yellow fever virus
ZIKV	Zika virus
